# The impact of a weight reduction program with and without meal-replacement on health related quality of life in middle-aged obese females

**DOI:** 10.1186/1472-6874-14-45

**Published:** 2014-03-12

**Authors:** Sadaf Koohkan, Denise Schaffner, Brandy J Milliron, Ingrid Frey, Daniel König, Peter Deibert, Mara Vitolins, Aloys Berg

**Affiliations:** 1 Department of Nutrition, Institut für Sport und Sportwissenschaft der Universität Freiburg, Schwarzwaldstrasse 175, Freiburg D-79117, Germany; 2 Department of Rehabilitative und Präventive Sportmedizin, Medizinische Universitätsklinik Freiburg, Universitätsklinikum Freiburg, Freiburg, Germany; 3 Department of Social Sciences & Health Policy, Division of Public Health Sciences, Wake Forest University Health Sciences, Winston-Salem, North Carolina; 4 Department of Epidemiology & Prevention, Division of Public Health Sciences, Wake Forest University Health Sciences, Winston-Salem, North Carolina

**Keywords:** Soy-based meal-replacement, Health-related quality of life, Obesity treatment, Weight loss intervention, Lifestyle intervention

## Abstract

**Background:**

In addition to an increased risk for chronic illnesses, obese individuals suffer from social stigmatization and discrimination, and severely obese people may experience greater risk of impaired psychosocial and physical functioning. Lower health-related quality of life (HRQOL) has been reported among obese persons seeking intensive treatment for their disease. To aid in the treatment of obesity, meal replacements have been recommended as an effective therapeutic strategy for weight loss, particularly when consumed in the beginning of an intervention. Hence, the objective of this study was to assess the impact of two 12-month weight reduction interventions (one arm including a meal replacement) on changes in HRQOL among obese females.

**Methods:**

This controlled trial compared two versions of a standardized 12-month weight reduction intervention: the weight-reduction lifestyle program without a meal replacement (LS) versus the same lifestyle program with the addition of a soy-based meal replacement product (LSMR). 380 women (LS: n = 190, LSMR: n = 190) were matched by age, gender, and weight (51.4 ± 7.0 yrs., 35.5 ± 3.03 kg/m^2^). This sample of women all completed the 12-month lifestyle intervention that was part of a larger study. The lifestyle intervention included instruction on exercise/sport, psychology, nutrition, and medicine in 18 theoretical and 40 practical units. Led by a sport physiologist, participants engaged in group-based exercise sessions once or twice a week. To evaluate HRQOL, all participants completed the SF-36 questionnaire pre- and post-intervention. Anthropometric, clinical, physical performance (ergometric stress tests), and self-reported leisure time physical activity (hours/day) data were collected.

**Results:**

The LSMR sample showed lower baseline HRQOL scores compared to the LS sample in six of eight HRQOL dimensions, most significant in vitality and health perception (p < 0.01). After the intervention, body weight was reduced in both lifestyle intervention groups (LS: -6.6±6.6 vs. LSMR -7.6±7.9 kg), however, weight loss and HRQOL improvements were more pronounced in the LSMR sample (LSMR: seven of eight, LS: four of eight dimensions).

**Conclusions:**

Our results show that HRQOL may improve among middle-aged obese females during a standardized lifestyle weight reduction program and may be enhanced by consuming a soy-based meal replacement product.

**Trial registration:**

ClinicalTrials.gov NCT00356785

## Background

Obesity is a serious chronic disease that is associated with a reduced life span, increased disability, and an increased risk for many serious illnesses such as cardiovascular disease, type 2 diabetes mellitus, and certain cancers [1]. In addition, obese individuals suffer from social stigmatization and discrimination, and severely obese people may experience greater risk of impaired psychosocial and physical functioning [2]. Thus, it is not surprising that various studies have found an association between obesity and poor cognitive performance [3-5] as well reduced quality of life [6-13]. Health-related quality of life (HRQOL) is assessed by measuring bodily pain, general health perception, and vitality [14,15]. Studies have reported a pronounced reduction in HRQOL among younger subjects and females [16-18]. A lower HRQOL has also been found among obese persons seeking intensive treatment for their disease [16,17,19].

There has been an ongoing interest in the HRQOL of obese persons [6,15]. The relationship between BMI and generic HRQOL has been examined through studies in Sweden [6], the United States [7,8], England [9], Australia [10,20] and France [13]. A consistent finding in these studies is that higher BMI is associated with lower HRQOL scores, particularly for the physical aspects of quality of life. Similarly, obesity-specific quality of life (assessed by the Obesity Specific Quality of Life Scale) has been reported to decrease as BMI increased in a population study in France [13]. Studies have reported that most domains of HRQOL were impaired in obese subjects, and more severely in younger subjects and in females [16,18,21,22]. Certain psychological problems, including binge-eating disorder and depression, are more common in obese persons as well [16,19,21,23].

The prevalence of overweight is rising worldwide despite increased spending on weight loss programs. There is agreement in therapeutic recommendations that people who are overweight and obese need to be counseled and given perspective and practical strategies for lifestyle changes. There is evidence that greater initial weight loss enhances the success of and the adherence to weight loss programs. Further, the use of meal replacements has been accepted as an effective therapeutic strategy for weight loss, particularly in the beginning of an intervention [24-26].

We have previously reported on the benefit of a meal replacement in weight reduction in combination with changes in atherogenic and diabetogenic measures, all very encouraging [27,28]. For meal replacement in this study, the formula which used was a protein-rich soy-based supplement which was specifically selected because of its low glycemic index [26-30].

The objective of the present investigation is to determine whether HRQOL changed in women before and after the 12 month lifestyle intervention program. We hypothesized that all participants who completed the lifestyle weight loss program would report improvement in HRQOL. Further, we expected that woman who completed the lifestyle program and consumed the soy-based meal replacement would report significantly higher improvements in HRQOL when compared to participants who did not consume a meal-replacement.

## Methods

### Study overview

This non-randomized, controlled trial tested the differences in HRQOL among two age- and weight-matched female subgroups of a larger study [31]. One group received the weight-reduction lifestyle program without a meal replacement (LS) and the other group received the same lifestyle program with the addition of a soy-based meal replacement product (LSMR).

### Participants and recruitment

Participants [31] were placed into training groups which included 15 to 18 patients. Obese adults were enrolled in this 12-month program if they had a BMI between 30 and 40 kg/m^2^ and had one or more obesity related comorbidity, such as hypertension, insulin resistance, or dyslipidemia. Participants had to be capable to be physically active (asymptomatic performance of at least 1 Watt per kg body weight and adequate motoric competence). The exclusion criteria included contraindications to physical exertion and currently in a weight reduction or calorie restriction program, type 1 diabetes, liver and kidney damage with the indication for restricted protein intake, psychiatric and eating disorders (bulimia, bulimia nervosa, binge eating disorder), intake of anti-obesity drugs, and status after malignant disease with freedom from disease of less than five years [31]. For the present investigation, 380 women who completed the intervention were matched by age and body weight (middle-aged females, 40 to 65 yrs., BMI 30 to 40 kg/m^2^). All females were of Caucasian race and 74% reported higher educational attainment.

### Lifestyle program

As central themes, exercise/sport, psychology/instruction, nutrition, and medicine were communicated in 18 theoretical and 40 practical units over a period of 12 months. Led by a sport physiologist, participants engaged in group-based exercise sessions once or twice a week. These sessions focused on endurance training (such as power or Nordic walking), as well as specific exercise to improve muscle strength, coordination and relaxation [31]. One of the goals of the intervention was to teach participants how to include exercise in their everyday lives. Behaviour change was discussed with a psychologist in the group meetings also. The participants received lifestyle counselling and brochures to address their barriers to participating in physical activity and also share their weight loss problems.

The nutritional counselling sessions were led by a dietician or nutritionist. Rather than strict diet plans, the program emphasized the importance of healthy food choices. The most important recommendations were to consume a low fat, carbohydrate consciousness, high protein diet. In addition, participants were allowed to take a meal replacement product as a supportive nutritional measure. For this, a clinically tested [26] and commercially available soy-yoghurt-honey product (Almased^®^) was recommended. The amount of protein in this product is 53.3% (83% soy-protein-isolate, and 17% milk protein). The product is characterized by favourable metabolic properties [30] such as a very low glycemic index (GI = 27), a very low glycemic load (GL of one consumption unit = 3.2), a low caloric content (354 kcal/100 g) [30], and biologically available isoflavonoids [32]. As described recently [27], the use of the product for meal replacement of obese subjects was recommended twice a day within the first six weeks (75 g per meal), and once a day in the following weeks. In our study the participants who requested Almased (almost 20% of the large study population) took it in the beginning of the program for no longer than three months; most of them (80%) during the first 12 weeks of intervention.

### Health-Related Quality of Life (HRQOL)

Health-related quality of life was assessed using the SF-36 questionnaire form [15,33,34] at baseline and after 12 months of intervention. The SF-36 questionnaire was used as a valid and reliable health indicator that encompasses emotional, physical, social and subjective feelings of well-being [15,35].

### Additional outcome measures

In addition to HRQOL, all outcome measures were assessed at baseline and at 12 months. Case Report Forms (CRFs) were completed by the study physicians. Physical activity was measured using the Freiburg Leisure Time Activity Questionnaire [36], and physical performance was tested using a standardized ergometric stress test [37]. Participant weight was measured using standardized methods by the study physician. All participants started the program after providing written informed consent. They received no incentives for participation and no incentives were provided for their weight loss success. Agreement of the Ethics Committee of the Freiburg University was received before the program was delivered [31]. The trial was registered as ClinicalTrials.gov NCT00356785.

### Statistical analyses

The data were analyzed using Statistical Package for Social Sciences (SPSS), version 13.0. The Wilcoxon test for paired samples was used for the intra-individual comparison between baseline and post intervention. Variance analysis was performed to establish whether there were significant differences between the subgroups with respect to the differences before and after the intervention. To verify the relationship between obesity, physical activity, performance and health related quality of life, a multiple regression analysis was applied. The differences between the initial and final state of the parameters such as BMI, total activity and weight-related performance presented in this case the independent variable X. The dependent variable Y was represented by each of the change in the quality of life in a SF36 scale. Spearman’s correlation coefficients were performed and statistical significance was defined as p < 0.05.

## Results

The characteristics of participants by group are displayed in Table 1. As noted, both groups were comparable at baseline in their anthropometric and clinical data (Table 1). No significant group differences were reported at baseline in physical fitness, measured as ergometric performance (Watt/kg body weight), and leisure time physical activity (hours/week). Females taking the meal replacement product (LSMR) reported lower baseline HRQOL scores than the LS subgroup. This difference was significant in six of eight HRQOL dimensions and was most pronounced in the scores for vitality and health perception (Table 2).

**Table 1:** Personal and anthropometric data of the females examined (mean ± SD)

**Anthropometric data in the total group and the two subgroups (LS; LSMR) before Intervention**			
	**Total group**	**Subgroup LS**	**Subgroup LSMR**
Number (n)	380	190	190
Age (yrs)	51.4 ± 7.0	51.2 ± 7.0	51.5 ± 7.0
Height (cm)	165.6 ± 6.0	165.6 ± 6.2	165.5 ± 5.7
Weight (kg)	97.4 ± 10.8	97.4 ± 10.8	97.4 ± 10.9
BMI (kg/m^2^)	35.50 ± 3.00	35.46 ± 2.98	35.52 ± 3.03
Physical performance capacity (watt/kg)	1.31 ± 0.30	1.31 ± 0.33	1.31 ± 0.27

**Table 2:** Anthropometric status, physical performance and SF36 dimensions according to the lifestyle intervention groups with and without meal replacement

	**Lifestyle intervention without meal replacement**				**Lifestyle intervention with meal replacement**			
**Measure**	**Baseline**	**12-month follow-up**	**Mean difference**	**Pre-Post difference (p-value)**	**Baseline**	**12-month follow-up**	**Mean difference**	**Pre-Post difference (p-value)**
Body weight (kg)	97.4 ± 10.8	90.8 ± 12.1	-6.6 ± 6.6	0.000	97.4 ± 10.9	89.8 ± 12.7	-7.6 ± 7.9	0.000
Body mass index (BMI; kg/m^2^)	35.5 ± 2.98	33.1 ± 3.63	-2.4 ± 2.34	0.000	35.5 ± 3.03	32.8 ± 4.12	-2.8 ± 2.78	0.000
Physical performance (watt/kg)	1.31 ± 0.33	1.51 ± 0.38	0.20 ± 0.27	0.000	1.31 ± 0.27	1.52 ± 0.41	0.23 ± 0.36	0.000
Sport activity (h/week)	1.37 ± 2.12	3.10 ± 2.80	1.73 ± 2.59	0.000	1.30 ± 2.13	2.88 ± 2.39	1.57 ± 2.83	0.000
Total leisure time activity (h/week)	6.41 ± 5.79	8.79 ± 5.33	2.38 ± 4.28	0.000	6.91 ± 6.10	9.32 ± 5.90	2.42 ± 4.32	0.000
Physical function	79.8 ± 13.95 **a**	88.5 ± 12.77	8.9 ± 14.66	0.000	76.2 ± 15.49 **a**	86.3 ± 16.68	10.3 ± 18.10	0.000
Functional role	82.5 ± 31.85	85.3 ± 31.39	3.6 ± 41.24	0.273	79.8 ± 30.84	84.7 ± 31.09	5.6 ± 40.14	0.048
Body pain	73.3 ± 23.94 **a**	76.4 ± 26.87	3.7 ± 25.87	0.052	67.9 ± 24.90 **a**	74.4 ± 25.34	6.2 ± 27.43	0.001
General health	65.8 ± 16.32 **a**	74.1 ± 16.19	9.1 ± 17.20	0.000	61.4 ± 16.21 **a**	70.8 ± 16.84	9.4 ± 17.90	0.000
Vitality	57.2 ± 15.97 **b**	65.1 ± 17.30	8.4 ± 17.79	0.000	52.4 ± 17.26 **b**	63.3 ± 16.09	11.0 ± 18.76	0.000
Social function	86.4 ± 18.23 **a**	87.7 ± 19.49	2.1 ± 23.30	0.164	81.5 ± 21.48 **a**	85.8 ± 18.47	4.4 ± 26.27	0.020
Role-emotional	85.3 ± 29.90	83.7 ± 33.19	0.4 ± 37.40	0.990	80.2 ± 34.07	84.3 ± 31.67	4.8 ± 36.99	0.086
Mental health	72.1 ± 15.70 **a**	75.0 ± 15.92	3.6 ± 14.91	0.001	69.3 ± 14.55 **a**	74.0 ± 15.32	4.8 ± 16.78	0.000
Physical component summary	48.2 ± 7.43	51.5 ± 8.19	3.3 ± 5.24	0.000	46.6 ± 8.10	50.7 ± 8.82	4.1 ± 7.42	0.000
Mental component summary	50.2 ± 9.33	50.7 ± 10.11	0.5 ± 1.59	0.145	48.7 ± 9.72	50.3 ± 9.80	1.6 ± 5.68	0.010

**a**: p < 0.05 **b**: p < 0.01 between group significance.

After 12 months of the intervention, body weight was reduced in both groups (LS: -6.6±6.6 p < 0.001 vs. LSMR -7.6±7.9 kg; p < 0.001). Weight reduction was more pronounced (p = 0.1) in the females taking the soy-based meal replacement product. However, lifestyle behaviour expressed by physical fitness (Watt/kg body weight) and leisure time physical activity (hours of physical activity per week) increased similarly in both groups (Table 2).

**Figure 1:**
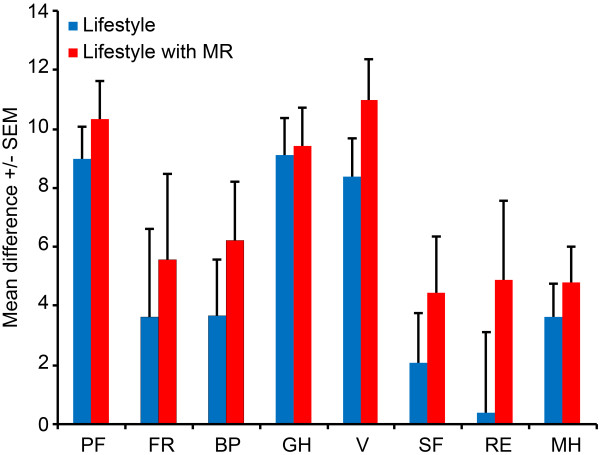
**Pre-post intervention differences in SF36 dimension (LS: Blue column, LSMR: Red column).** For vitality (V) and role-emotional (RE) there is a trend for significance (p < 0.1) in intra-group comparison. **PF**: Physical Function, **FR**: Functional Role, **BP**: Body Pain, **GH**: General Health, **V**: vitality, **SF**: social Function, **RE**: Role-Emotional, **MH**: Mental Health.

HRQOL increased in both groups after 12 months. Four of the eight HRQOL dimensions significantly improved in the LS group (physical function (p = 0.000), general health (p = 0.000), vitality (p = 0.000), mental health (p = 0.001)); and seven of the eight HRQOL dimensions improved significantly in the LSMR group (physical function (p = 0.000), functional role (p = 0.048), body pain (p = 0.001), general health (p = 0.000), vitality(p = 0.000), social functioning (p = 0.020) and mental health (p = 0.000)) (Table 2). In addition, the physical component summary significantly improved in both groups after the intervention (p = 0.000), whereas the mental component summary increased in the LSMR group only (p = 0.010) (Table 2). When intra-group comparison was done, there is a trend of significance (p = 0.1) for changes vitality and role emotional (Figure 1), however only the mental component summary reaches the statistical significance level (p = 0.01) showing a benefit for the LSMR group. In both groups the correlation between healths related quality of life items were increased after intervention particularly in items of physical functions. However, the biological consequence of these correlations may be small (less than 10% of the variance) (Table 3).

**Table 3:** Multiple regression function between life style factors (x: BMI, physical performance, leisure time physical activity) and the 8 items of HRQOL (y) in the groups investigated

**Variables**	**Lifestyle intervention without meal replacement**		**Lifestyle intervention with meal replacement**	
	**r (at baseline)**	**r (post interv.)**	**r (at Baseline)**	**r (after interv.)**
Physical function	0.219 _**b**_	0.319 _**c**_	0.282 _**c**_	0.361 _**c**_
Functional role	0.070 _**n.s**_	0.179 _**a**_	0.131 _**n.s**_	0.279 _**c**_
Body pain	0.064 _**n.s**_	0.202 _**b**_	0.112 _**n.s**_	0.232 _**b**_
General health	0.126 _**n.s**_	0.282 _**c**_	0.048 _**n.s**_	0.279 _**c**_
Vitality	0.112 _**n.s**_	0.260 _**c**_	0.156 _**a**_	0.238 _**c**_
Social function	0.071 _**n.s**_	0.133 _**n.s**_	0.167 _**a**_	0.130 _**n.s**_
Role-emotional	0.040 _**n.s**_	0.143 _**a**_	0.058 _**n.s**_	0.065 _**n.s**_
Mental health	0.070 _**n.s**_	0.198 _**b**_	0.150 _**a**_	0.108 _**n.s**_

**r**: coefficient of correlation; **n.s**: not significant, **a**: p < 0.05, **b**: p < 0.01, **c**: p < 0.00.

## Discussion

In contrast to baseline values, both groups showed comparable values in all HRQOL dimensions after the intervention. In addition, both groups reported HRQOL score values after the intervention that compare to those reported in other middle-aged female populations [38,39]. Despite successful changes in lifestyle dimensions, the changes in the HRQOL scores were not significantly correlated with the corresponding changes in anthropometric measures, fitness data, individual changes in body weight, or individual increases in leisure time physical activity. Further, the success of the intervention in our sample did not depend on age or initial BMI values.

The obese middle-aged women in this study were successful in losing weight in a standardized interdisciplinary program which focused on modifying dietary and physical activity [31]. We hypothesized that all participants who completed the lifestyle weight loss program would also report improvement in HRQOL. Further, we expected that woman who completed the lifestyle program and consumed the soy-based meal replacement would report significantly higher improvements in HRQOL when compared to participants who did not consume a meal-replacement. Over a period of 12 months, most of the participants were able to lose 5 to 10% of their initial body weight and increased their physical fitness and leisure time physical activity by more than two hours per week. As expected, and in agreement with other intervention studies, these lifestyle changes were positively accompanied with changes in physical and mental well-being. At the end of the 12 months intervention, both groups showed promising nutrition and physical activity behaviours that have been linked to weight maintenance [40].

In agreement with the results of representative studies and reviews, we also found that the baseline impairments in the SF-36 in both groups were predominantly focused on physical functioning, including general health, vitality and bodily pain. However, our findings suggest that impairments could also be found in the mental health. Assuming that our middle-aged obese females were suffering from some chronic diseases such as arthritis, back pain, hypertension and/or diabetes, it may be difficult to differentiate between the attributions of significant factors responsible for the HRQOL impairments. There is agreement that chronic illness may impair HRQOL in obese persons, whereas physical activity and physical fitness may improve HRQOL in obese subjects [21]. In our sample of middle-aged females, obesity, physical fitness, and leisure time physical activity could not explain impaired HRQOL dimensions. Similarly, research including large samples of differing gender, race, BMI and age, reported that only 28% of the variance in obesity-specific HRQOL could be explained by increases in BMI, whereas 72% of the variance remained unexplained [21].

It has been well documented that obesity-specific HRQOL is significantly more impaired in subjects seeking intensive weight-loss treatment. For example, gastric bypass patients have shown the most impairment and the poorest HRQOL among obese subgroups [21]. When the SF-36 scores measured in the two subgroups of our sample were compared, the females showing more significant impairment in their SF-36 scores were those who consumed the soy-based meal replacement product within the first months of the weight loss program. The two intervention groups in this study were comparable by age, BMI, physical fitness, anthropometric and clinical measures, suggesting that there may be a subgroup of females who seek more intensive treatment characterized by poorer HRQOL. Women included in this subgroup may need a more intensive and individualized treatment including psychological supervision and clinical support.

Initial weight loss supports individual willingness to continue an ongoing weight loss program and reflects the biological effectiveness of a structured therapeutic program [31,41-44]. For example, in our study the group taking the soy-based diet product benefited by its use as a meal replacement during their weight reduction program throughout the 12 month intervention. These females started with lower HRQOL score and reached normal scores within the intervention. They also lost more weight compared with the group who received the standardized lifestyle program without the meal replacement. The data we present suggests that obese females participating in the weight reduction training program who started at a lower score of quality of life, requested therapeutically advice in addition to the standardized program, e.g. use of meal replacement product. Additionally, independent of changes in body weight, physical fitness and leisure time physical activity, the success of the intervention may be influenced by the dietary regimen (e.g. intake of low-glycemic and protein-rich foods) [45,46]. Recently published results showed that weight loss and lifestyle interventions were associated with HRQOL in patients with Type 2 Diabetes Mellitus (T2DM) [47]. Other research has reported associations between high protein diets and improvements in body weight and metabolic risk factors; and between soy protein and improvements in cognitive function and mood, brain function and memory [46,48-53].

As a result of their molecular similarities to endogenous estrogens, soy isoflavones may induce estrogenic effects by interaction with the estrogens receptors in various tissues [53], particularly in the nervous system by inhibition of tyrosin kinase and protective effects on neurotransmitter systems [54]. However, further research is needed to understand the efficacy of isoflavones on the nervous system and their possible role in mental function, and last but not least on quality of life. Finally, our results are parallel with the findings of the BLISS Study *(Behavioural Lifestyle Intervention for Savvy Survivors)* which reported a positive effect of a soy-protein-rich supplement used in females after breast cancer treatment. Vitolins et al. (under review, 2013) reported improvements in HRQOL in breast cancer survivors participating in a weight loss intervention that used a soy-based meal replacement. Participants replaced one or two meals each day for 12 weeks, in addition to exercise and cognitive behavioral therapy. With the exception of breast concerns, all aspects of Quality of Life, as measured using the Functional Assessment of Cancer Treatment – Breast (FACT-B), were improved at the end of the 12-week intervention.

## Conclusions

In conclusion, we found significant changes in HRQOL in a large, age and BMI matched sample of women who completed a 12-month weight reduction intervention. In addition, participants who consumed the soy-based meal replacement product showed a trend for greater weight loss and improvement in HRQOL. Small changes to replace one meal or snack with a healthier, lower calorie option may promote long-term weight changes. Strengths of this study include the large study population. Limitations in this study include data collection in a non-randomized sample and the inability to determine exactly which component of lifestyle change most impacts HRQOL. Therefore, additional clinical and research efforts should focus on specific markers of psycho-social and psycho-mental variables during and after weight loss. In addition, it is necessary to identify persons who may require intensified treatment in weight loss strategies (e.g. meal replacement). Future studies should investigate which factors, other than weight loss and increased physical activity, may be responsible for the improvements in HRQOL in obese middle-aged women participating in weight reduction program.

## Competing interests

Research related of this study was funded by the Almased GmbH, Bienenbüttel, Germany, and A. Berg has written “The Almased Wellness Concept” outlining the effects of a soy-enriched diet in health and disease.

## Authors’ contributions

SK: Writing of the manuscript, conceive and coordination, interpretation of data. DS: Data analysis and interpretation of data. BM: interpretation of data and editing the manuscript. IF: Data preparation and statistical analysis. DK: Study GP, supervision of the participants during the study, data acquisition. PD: Study GP, supervision of the participants during the study, data acquisition. MV: interpretation and final discussion of data. AB: Project leader, design of study, conceived of the study, interpretation of data, guiding and supervising of manuscript writing, decision to submit. All authors read and approved the final manuscript.

## Pre-publication history

The pre-publication history for this paper can be accessed here:

http://www.biomedcentral.com/1472-6874/14/45/prepub
